# The effect of cationically-modified phosphorylcholine polymers on human osteoblasts in vitro and their effect on bone formation in vivo

**DOI:** 10.1007/s10856-017-5958-8

**Published:** 2017-08-17

**Authors:** Jonathan M. Lawton, Mariam Habib, Bingkui Ma, Roger A. Brooks, Serena M. Best, Andrew L. Lewis, Neil Rushton, William Bonfield

**Affiliations:** 1 Department of Materials Science and Metallurgy, Cambridge Centre for Medical Materials, University of Cambridge, New Museum Site, Cambridge, CB2 3QZ UK; 2 Orthopaedic Research Unit, University of Cambridge, Addenbrookes Hospital, Hills Road, Cambridge, CB2 2QQ UK; 3 Biocompatibles UK Ltd, Chapman House, Farnham Business Park, Weydon Lane, Farnham, Surrey, GU9 8QL UK

## Abstract

**Abstract:**

The effect of introducing cationic charge into phosphorylcholine (PC)-based polymers has been investigated in this study with a view to using these materials as coatings to improve bone formation and osseointegration at the bone-implant interface. PC-based polymers, which have been used in a variety of medical devices to improve biocompatibility, are associated with low protein adsorption resulting in reduced complement activation, inflammatory response and cell adhesion. However, in some applications, such as orthopaedics, good integration between the implant and bone is needed to allow the distribution of loading stresses and a bioactive response is required. It has previously been shown that the incorporation of cationic charge into PC-based polymers may increase protein adsorption that stimulates subsequent cell adhesion. In this paper, the effect of cationic charge in PC-based polymers on human osteoblasts (HObs) in vitro and the effect of these polymers on bone formation in the rat tibia was assessed. Increasing PC positive surface charge increased HOb cell adhesion and stimulated increased cell differentiation and the production of calcium phosphate deposits. However, when implanted in bone these materials were at best biotolerant, stimulating the production of fibrous tissue and areas of loosely associated matrix (LAM) around the implant. Their development, as formulated in this study, as bone interfacing implant coatings is therefore not warranted.

**Graphical abstract:**

## Introduction

Advances in modern medicine, such as the introduction of penicillin, antiseptics, and vaccinations, have significantly increased human life expectancy [1]. The ageing population in the West (in the UK and US alone there are over 100 million individuals over the age of 50) [2], coupled with the deterioration of bone stock with age (particularly for woman of post-menopausal age) [3], mean that the clinical need for tissue repair and replacement has never been greater. Bone tissue loss, predominantly due to degenerative diseases (such as osteoporosis), osteosarcoma and trauma [4], has led to the design of a variety of surgical techniques, medical devices and specialised materials. These range from total hip and knee replacements to fracture fixation devices and bone stock replacements.

Phosphorylcholine (PC) materials are bio-inspired polymers that mimic the extracellular surface of red blood cells, containing an exact chemical copy of the predominant zwitterionic phospholipid headgroup found in the cell lipid membrane. Unlike most biomaterials, the well-hydrated and neutrally-charged PC surface allows for the interaction of proteins without inducing shape changes in the protein’s three-dimensional structure and thus reduce irreversible protein adsorption [5]. Furthermore, this decrease in protein adsorption results in decreased blood clotting [6], cellular adhesion, and in a reduction in the inflammatory response and fibrous capsule formation [7]. Such properties have resulted in PC materials being used for a variety of biomedical applications where a passive interaction between the material and the body is required. Examples include coating blood contact devices, such as coronary guide wires [8] and stents [9], extracorporeal circuits [10], and for contact lenses [11]. PC materials have also found utility in the orthopaedic field, as superlubricious, low-wear surfaces grafted onto polyethylene acetabular liners [12], for which there are now 3 year data from an 80 patient study [13], colbalt-chromium-molybdenum metal alloy bearings [14, 15] and poly(ether-ether ketone) orthopaedic bearing surfaces [16].

However, in some clinical applications a “bioinert” response is not the most appropriate. For example, orthopaedic implants, such as artificial hips, require a degree of interaction between the femoral stem and the surrounding bone tissue in order to enhance load transfer and to decrease micromotion and stress shielding of the implant, which can lead to implant loosening and premature failure [17, 18]. Furthermore, increasing the speed and quantity of bone-implant integration allows early loading, resulting in quicker patient mobility, improved patient well-being, shorter hospital stays, and a reduction in healthcare costs [1]. An important development in this regard was plasma-sprayed hydroxyapatite coatings which produce improved bone bonding of implants to bone and increased longevity of some joint replacements [19, 20]. However, the risks associated with these relatively thick coatings is delamination and there is continued concern about the resorption of HA by interfacial remodelling in some applications [21–23]. The phosphorylcholine side chain of the PC-polymer contains the phosphate moiety present in phosphatidylcholine and phosphatidylserine, the latter being membrane lipid component that is able to bind calcium and is implicated in biomineralization [24–26]. This suggested the potential of PC polymer to also interact with calcium and form a stable interface with bone mineral. However, osteoblast activity at the interface would be required for osteointegration and unmodified PC polymer has reduced cellular interaction. Attempts have been made to modify PC polymers to produce materials with inherent biocompatibility but also with additional components to invoke specific biological interactions. In one study, a PC-containing copolymer of N-isopropylacrylamide and N-(n-octadecyl) acrylamide was shown to preferentially adhere U937 macrophages, which subsequently increased expression of TNF-α in response to Co^2+^ and Co ^3+^ ions [27]. In another approach, researchers have also shown that the introduction of surface charge into biomaterials increases cell spreading, adhesion, growth and proliferation [28–31]. Rose et al [32] demonstrated that the introduction of a cationic moiety (choline methacrylate chloride salt – CMA) into PC polymers also significantly increased the amount of protein adsorption and subsequent cell attachment, where the amount of protein adsorption and cell attachment was found to be protein, cell and CMA monomer content specific. The authors [32] concluded that the increase in PC bioactivity was caused by the increase in the polymer surface charge, whereby the predominantly negatively charged biomolecules interact electrostatically with the positively charged surfaces. Moreover, Palmer et al [33] have shown that cationically- modified PC materials can also act as drug delivery vehicles, where negatively charged therapeutic biomolecules interact reversibly with PC’s positive charge and are released in a controlled manner. In a comparative study of PC and cationically-modified PC coatings on titanium porous oxide surface implants placed in trabecular and cortical bone of rabbits [34], although there was no significant differences in bone density between the coatings or uncoated control, there was a higher bone-implant contact for the cationically-charged PC coating and uncoated control compared to the PC coating (*p* < 0.05) at the 6 week explant point. This somewhat indicates that the surface charge is able to orchestrate cellular interaction, overcoming the inherent anti-adhesiveness of the PC material, but only histometric and biomechanical analysis was performed and hence no mechanism proposed. The aim of current work presented herein was to investigate the effects of high and low charge levels of cationically-charged PC materials on human osteoblast cells (HObs). Osteoblasts are skeletal cells responsible for bone formation [35]. They are derived from messenchymal stem cells found in the bone marrow [36] via osteoprogenitor cells and they secrete extracellular matrix (type I collagen) and non-collagenous material that is involved in the nucleation and formation of bone mineral [37]. Hence the cells are likely being influenced either directly or indirectly by the implant surface characteristics. We describe in vitro experiments in order to shed light on potential mechanisms of interaction between biomaterial and tissue and investigate the in vivo longevity of any beneficial effect in a 14 week rat tibia implantation model.

## Materials and methods

### In vitro experiments

#### Materials

**Fig. 1:**
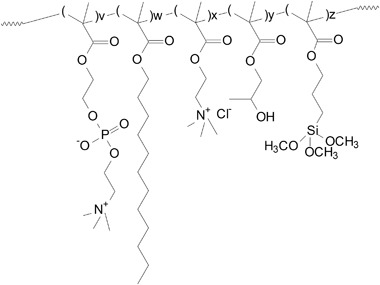
A schematic chemical representation of a cationically charged phosphorylcholine polymer

The PC polymers used in this experiment have previously been characterised [38]. They are methacrylate-based polymers synthesised using free radical polymerisation of 2-methacryloxyethylphosphorylcholine (MPC), laurylmethacrylate (LMA), hydroxypropylmethacrylate (HPMA) and trimethoxysilylmethacrylate (TMSMA). Positive charge was introduced by including choline methacrylate (CMA). A schematic representation of this polymer is shown in Fig. 1 and the constituents (weight percentages) and their function are detailed in Table 1. In addition to the other polymers, PC5 was used in the in vitro experiments and PC6 was used to coat the in vivo implants due to the availability of these two substrates when these two experiments were carried out. The difference in response to the small difference in cationic charge between these two PC polymers is unlikely to be significant. The polymers were supplied by Biocompatibles UK Ltd (Farnham, Surrey, UK).

**Table 1:** Constituents of the phosphorylcholine materials (weight percent)

		Monomer content (wt%)				
Monomer		PC20	PC6	PC5	PC0	Component function
MPC	v	22	29	28	29	Phosphorylcholine head group—charge neutrality—non-thrombogenic
LMA	w	41	50	50	51	Lauryl group—alkyl chain component—hydrophobic—substrate adsorption
HPMA	x	12	12	12	15	Aids crosslinking and film formation
TSMA	y	5	4	5	5	Methoxysilyl crosslinker—mechanical stability. Aids substrate adhesion
TMA	z	20	6	5	0	Cationic charge

Each PC polymer was dissolved in ethanol at a concentration of 10 mg/ml and the solution filtered through a 0.2 μm filter. Tissue culture plates and flasks were then filled with the polymer-ethanol solution and left for 5 min at room temperature before being emptied. After air-drying for 2 h at room temperature, the polymers were cured onto the plates and flasks at 70 °C for 72 h. The coated culture dishes were then sterilised under UV light for 2 h.

#### Cell culture

Human osteoblasts (HObs) (PromoCell, Heidelberg, Germany) isolated from hip bone were grown in 75 cm^2^ tissue culture flasks in McCoy’s 5a growth medium (Invitrogen, Paisley, UK) containing 10% foetal bovine serum (FBS), 1% glutamine and 30 μg/ml vitamin C (standard culture conditions) (Sigma, Poole, Dorset, UK). After cryogenic recovery and 24 h incubation, cell adherence was checked and the medium replaced; thereafter the medium was replaced every 2 days. At 60–80% confluence, the cells were washed three times with Hank’s Balanced Salt Solution (HBSS) (Invitrogen) and incubated (RT for 5 min) before trypsin/ethylenediaminetetraacetic acid (EDTA) (Sigma) was used to detach the cells from the flask. Complete cellular detachment was confirmed by phase-contrast microscopy. After 5 min, McCoy’s growth medium was added to neutralise the trypsin/EDTA. The cell suspension was then centrifuged at 220 × g for 4 min at room temperature before the supernatant was aspirated and the cell pellet re-suspended in 1 mL of McCoy’s medium and counted using a haemocytometer.

#### Cell number

HOb cells were cultured on non-coated, PC5 and PC20 coated T25 flasks for 28 days in medium (as previously described), incubated at 37 °C in 5% CO_2_ humidity. Cell number was determined at 6 h, 1, 2, 4, 7, 14 and 28 days using Vialight^TM^ HS proliferation/cytotoxicity kit (Lonza, UK). In this assay, luciferase enzyme reacts with adenosine trisphosphate (ATP) (found in all metabolically active cells) to emit light and its intensity is indicative of cell number. At each time point the culture media was aspirated and the cells washed 3 times with phosphate buffer saline solution (PBS) (Sigma). For each flask 1 mL 0.5% v/v Triton X-100 (VWR, Lutterworth, UK) in PBS was added and two freeze thaw cycles (15 min at −70 °C and 37 °C) were used to lyse the cell suspension. 180 μL of the lysate was then transferred in duplicate to a white-walled 96-well plate and 20 μL of ATP monitoring reagent added to each well. The ATP concentration was then immediately read (TopCount.NXT^TM^, Packard BioScience Co, Meriden, USA). Using a standard curve (light intensities of pre-determined HOb cell numbers of 0, 2500, 5000, 10000, 20000 and 40000 on uncoated well plates) the cell numbers on the substrates was determined. Cell number was also determined for cells on PC0 at 6 h, 1 and 2 days. Three separate flasks were used for each substrate and time point.

#### Cell differentiation

HOb cells were cultured on non-coated, PC5 and PC20 coated T25 flasks for 28 days in McCoy’s medium. Cell differentiation was determined by identifying the enzyme alkaline phosphatase (ALP), a widely recognised [39] enzyme marker of osteoblast differentiation associated with skeletal mineralisation [40]. 0.5 M 2-amino-2-methyl-1-propanol (AMP) substrate buffer (Sigma) was prepared in distilled water (pH 10) and supplemented with 2 mM magnesium chloride (MgCl_2_) and 9 mM *p*-nitrophenol phosphate (*p*-NPP) (Sigma). In an alkaline solution, ALP catalyses *p*-nitrophenyl phosphate to *p*-nitrophenol, appearing yellow in colour. Cells were lysed at 1, 2, 4, 7, 14 and 28 days in 3 flasks per substrate per timepoint (as described for ATP measurement) and 50 μL of each lysate in duplicate and 50 μL of a range of *p*-nitrophenol concentrations, diluted to produce a standard curve were transferred to a 96-well plate. Subsequently, 50 μL of AMP buffer was added to each well and the plate incubated at 37 °C for 15 min. Absorbance was immediately read at 405 nm using an EL800 Universal Microplate reader (Bio-tek Instruments, Inc., Winooski, USA). A standard curve of absorbance as function of *p*-nitrophenol concentration was generated and used to determine the total ALP content of each test sample.

Optical Imaging and Energy Dispersive X-ray (EDX) Characterisation of Cells HOb cells were cultured (seeding density of 12 × 10^3^/cm^2^) in T25 flasks coated with PC0, PC5 or PC20 and on a non-coated tissue culture plastic control for 48 h and 28 days. Optical imaging using a Leitz Labovert phase contrast microscope was carried out on all substrates at 48 h. For SEM, the cells on the substrates at 48 h and 28 days, were washed with PBS, fixed in 4% paraformaldehyde (Sigma) and washed three times with distilled water. A square piece, approximately 20 × 20 mm, of each flask was cut and mounted onto an SEM stub using double-sided carbon tape and sputter coated with carbon. A JEOL XL30 scanning electron microscope (SEM) at 5 kV was used to image the samples and EDX was used to characterise the elemental constituents of mineral exudates on the cells and ECM (extracellular matrix).

### In vivo experiment

#### Materials

**Fig. 2:**
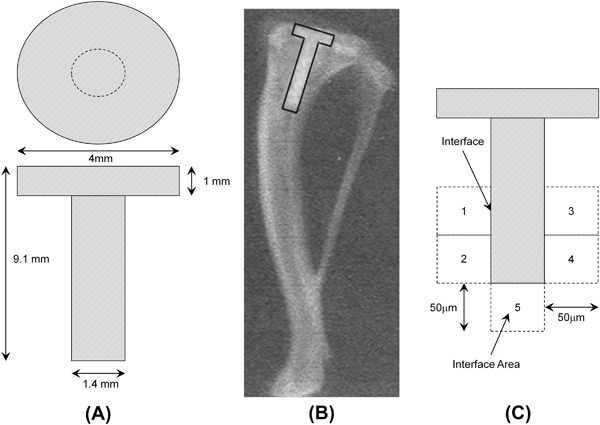
**a** Schematic representation of the stainless steel pins used for implantation; **b** a radiograph indicating the placement of the surgical pin in the rat tibia; **c** diagrammatic map of a laterel sectioned pin. The figure indicates the locations where images for histological analysis were captured (1–5) and the local distinction between the interface and interfacial area

Thirty surgical grade stainless steel (316 L) pins (R J Layland, Rayleigh, UK) were cleaned using dry Emory cloth paper (P1200, T444 Norton, Tufbak Durite) and sonicated for 5 min in both acetone and ethanol. The pins were equally assigned to one of five groups and coated accordingly. Six pins were uncoated (negative control). Six pins were plasma-spray coated with 60–100 μm of hydroxyapatite (Plasma Biotal, Tideswell, UK) (positive control). Three groups of six were then double dip coated at 4 mm/sec in 5 mg/ml polymer concentrations in ethanol of PC0, PC6 or PC20 respectively (as previously described in Table 1), resulting in polymer coatings approximately 30–50 nm thick. The polymer coated pins were then cured at 70 °C for 4 h. All pins were designed to have equal dimensions prior to implantation (Fig. 2a) and were sterilised using gamma irradiation (25 kGy, Isotron, UK).

#### Surgery

The surgical method for implantation has previously been described by Allen et al [41] and others [42, 43]. All procedures underwent ethical review and were carried out in accordance with the regulations as set out in the Animal (Scientific Procedures) Act 1986. Thirty mature Sprague Dawley rats, weighing between 300–350 g (Harlam UK Ltd, Bicester, UK) were randomly allocated to one of the five groups. The number of animals used per group was based upon previous studies which showed significant results with groups of 6. One pin was implanted into the right tibia of each animal (Fig. 2b).

All drugs were obtained from National Veterinary Services, Stoke-on-Trent, UK. Briefly, anaesthesisa was induced using a gaseous mixture of oxygen and halothane (4%), at a rate of 6 litres/min in an anaesthetic chamber. Midazolam (3 mg/kg) was given by intra-peritoneal injection and the rats maintained with halothane 4% delivered through an anaesthetic mask. The right hind limb was shaved, peri-operative analgesia administered sub-cutaniously (Rimadyl—carprofen, 5 mg/ml) and the rat placed supine. A 1.5 cm incision was made lateral to the patella and a lateral capsulotomy performed allowing medial dislocation of the patella exposing the tibial plateau. A 1.5 mm diameter hand drill was used to drill through the centre of the tibial plateau to a depth of 10 mm and counter sunk (using a 4 mm counter-bore) to allow the head of the implant to be flush with articulating surfaces. The pin was then press-fit into the tibia, the patella reduced and the incision closed. Immediately after surgery the rats received 150 mg/kg of antibiotic (Synulox—coamoxyclavulanic acid) intramuscularly and a sub-cutaneous injection of 0.15 mg/kg analgesia (temgesic: buprenorphine). The rats were allowed to recover in IVC caging systems (Techniplast UK Ltd, Northants, UK) and after 5 h a second injection of temgesic (0.1 mg/kg) was administered.

#### Tissue processing

The rats were sacrificed at 14 weeks according to Home Office (UK) schedule 1. The right tibia was removed and fixed in ice-cold paraformaldehyde in 0.1 M phosphate buffer (pH 7.4) with 0.1% w/v sucrose and 0.05% v/v gluteraldehyde at 4 °C. After 5 h the tibias were washed three times in PBS and dehydrated in ascending concentrations of methylated spirit (50–100%) before being de-fatted using continuously topped-up acetone under vacuum for 7 days. The tibias were then embedded in poly(methyl methacrylate) (PMMA). Methyl methacrylate (Sigma) containing 2.5% w/v benzoyl peroxide (catalyst) and 2.5% v/v dibutyl phthalate (plasticiser) was infiltrated into the specimens at 4 °C under vaccuum for 7 days. The methacrylate solution was then replaced with fresh solution and polymerised. Once polymerised, 300–500 μm thick longtitudinal sections (sagital plane of the pin and tibia) were cut using a diamond saw (100 CA blade 0.1 mm/sec, Accuton 5, Struers, Glasgow, UK) and glued (Cyno 40, Delta, Leeds, UK) onto frosted glass microscpe slides before being ground and polished to 100 μm thick using a graded series of silicon carbide papers (Struers, Denmark).

#### Microscopy and histological analysis

Slides were washed in liquid detergent and water for 1 min, rinsed in water for 3 min and stained with toluidine blue (pH 9, 56 °C, 30 min). Five fields (Fig. 2c) for each slide were digitally photographed using a Leitz Dialux 20 light microscope (X100) with attached camera and stored on a computer (Aquis Image Acquisition software—Synoptics, Cambridge, UK).

The head of the pin was ignored due to relatively poor bone apposition in this region. Such a response may be due to stress shielding (poor stress distribution through the pin) resulting in bone resorption rather than due to the problems associated with surgery and experimental technique and has been observed previously [33]. The image was then edited in Adobe Photoshop 6.0 and cropped to allow a distance of 50 μm from the pin surface into the surrounding bone and bone marrow leaving an image of the bone-implant interface (Figs. 2c and 8). Four tissue types, bone, marrow, fibrous tissue and loosely associated matrix (LAM), were then identified and colour coded. The coloured images were then processed into binary images. Scion Image Analysis (Scion Corporation, USA) was used to measure the areas of the four tissue types and these were expressed as a percentage of the total area for each field position on each individual pin (interfacial area). The percentage of each tissue was also calculated along the length of the interface between the pin and the bone (interface). The five positions along the pin were then summed and averaged.

#### Statistical analysis

For both the in vivo and in vitro experiments the data was compared using a one-way ANOVA. Identified trends were further investigated using either specific t-testing or Bonferonii analysis to highlight significant differences. The distribution of the data is represented using standard error.

## Results

### In vitro experiments

**Fig. 3:**
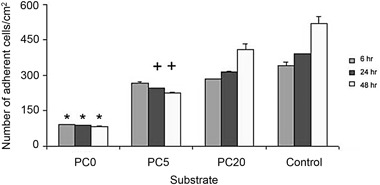
The effect of PC and cationically-modified PC on the number of HOb cells in vitro after 6, 24 and 48 h in culture as compared to control surfaces. *p < 0.001 compared to other materials at the equivalent time point. +*p* < 0.001 compared to PC20 and control at the equivalent time points

Figure 3 indicates the number of HOb cells on PC coated and control surfaces over 48 h. At each time point, tissue plastic control surfaces exhibited the largest number of cells. Significant differences (*P* < 0.001) were observed between the control and PC0 and between PC20 and PC0 surfaces at all three time points. However, significant differences (*P* < 0.001) between PC20 and PC5 were only seen at 24 and 48 h.

**Fig. 4:**
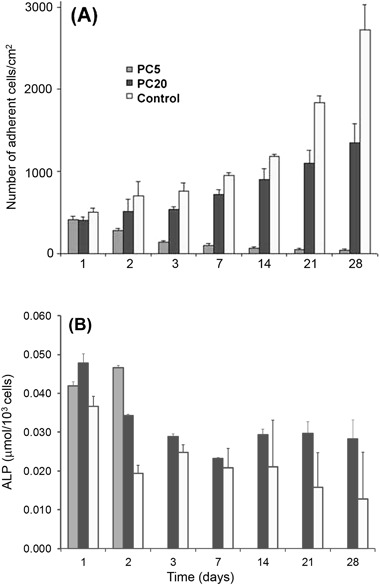
**a** The effect of cationically-modified PC on the number of HOb cells in vitro over a 28-day period in culture as compared to control surfaces. The number of cells on control surfaces was significantly greater (*p* < 0.001) than on PC20 and PC5 at all time points and on PC20 than PC5 after day1; **b** corresponding effect of cationically-modified PC on HOb cell ALP production in vitro over a 28-day period as compared to control surfaces

Figure 4a indicates the number of HOb cells on charge-modified PC coated and control surfaces over 28 days. The number of HOb cells increased with time on PC20 and tissue plastic control. Interestingly, the number of cells decreased on PC5 with increasing time, up to 28 days. After day 1, significant differences (*P* < 0.001) existed between PC5 and PC20 and control surfaces for all time points. This indicates that HOb cells proliferated well on PC20, however, proliferation on control surfaces was significantly greater at all time points (*P* < 0.001).

Figure 4b shows the effect of time and charged PC substrate on cell differentiation. The ALP activity per thousand cells decreased on PC20 over the first 3 days of culture and on the control substrate between day 1 and 2. Thereafter ALP activity did not change significantly and there was no significant difference between ALP activity on PC20 and control substrates after day 2. Alkaline phosphatase activity and the number of cells on PC5 was too low to give an accurate measure of normalised activity

**Fig. 5:**
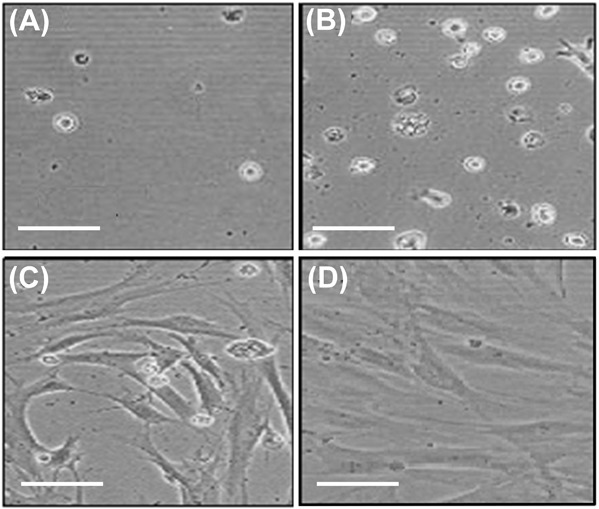
Photomicrographs of HOb Cells on **a** PC0, **b** PC5, **c** PC20 and **d** control surfaces at 48 h in vitro. (Scale bars = 100 μm)

Figure 4a shows the effect of PC charge content on HOb cell proliferation and spreading at 48 h and supports the data presented in Fig. 3. PC20 and the control surfaces showed the largest number of cells, and on these surfaces the HOb cells have developed fully-spread morphologies (Fig. 5). PC0 supported minimal cell adhesion and, although there were more cells on PC5, the cells on both these substrates remained rounded. The photomicrographs in Fig. 5 also indicate that the cells on tissue plastic controls and on PC20 showed the presence of mineral deposits.

**Fig. 6:**
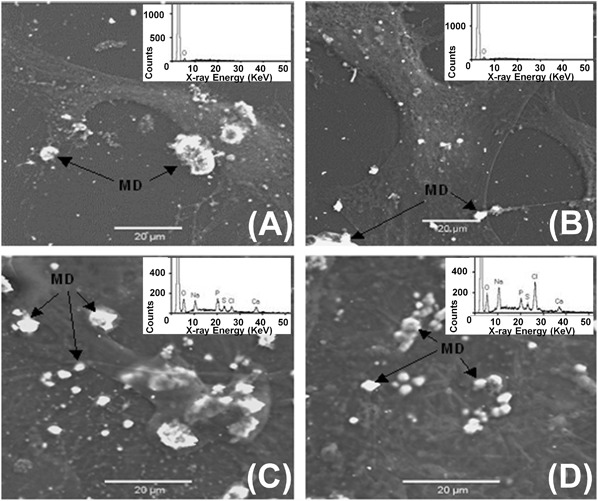
SEM images showing osteoblast cells on **a** PC20 and **b** control at 48 h and **c** PC20 and **d** control at 28 days. An increase in the amount of mineral deposits (MD) were seen on both PC20 (**c**) and control (**d**) surfaces at 28 days. Corresponding elemental analysis is shown in the inset for each image and demonstrates that these mineral deposits contain calcium and phosphorus

Figure 6 shows SEM with elemental analysis and supports Fig. 5, confirming both the presence of mineral deposits on PC20 and control surfaces at 48 h and that the number of these mineral deposits increased with time (up to 28 days). Figure 6 also shows that the mineral deposits on both PC20 and control surfaces at 28 days contained both calcium and phosphorus.

### In vivo results

**Fig. 7:**
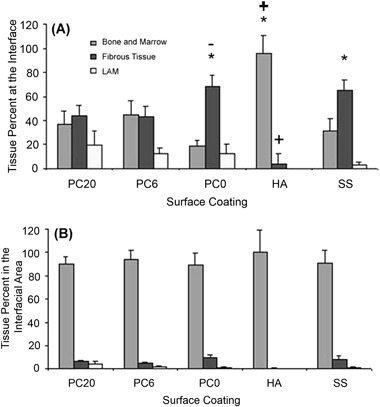
The effect of PC and cationically-modified PC on the percentage of bone and marrow, fibrous tissue and loosely associated matrix (LAM) compared to hydroxyapatite (HA) and stainless steel (SS) positive and negative controls at **a** the bone-implant interface. * Significantly more (*p* < 0.05) than the other two tissues on that material. +Signficantly different amounts of tissue on HA (*p* < 0.05) than the same tissue on the other 4 materials. **b** In the bone-implant interfacial area

Figure 7a indicates the amount (percentage) of different types of tissue at the bone-implant interface. For HA there was significantly (*p* < 0.05) more bone and marrow than both fibrous tissue and LAM. Both cationically charged PC materials produced a similar response, with no significant differences found between any of the tissue types. For both PC0 and SS there was significantly (*p* < 0.05) more fibrous tissue than both bone and marrow and LAM. For SS there was also significantly more bone and marrow than LAM. HA had the highest bone and marrow apposition compared to the other four surfaces and also less fibrous tissue (*p* < 0.05). In a comparison of the PC materials, PC0 had significantly (*p* < 0.05) more fibrous tissue than PC6 but there were no significant differences between PC0 and PC20 or between PC0 and SS. Furthermore, there was no significant difference between any of the PC groups in relation to the formation of LAM.

**Fig. 8:**
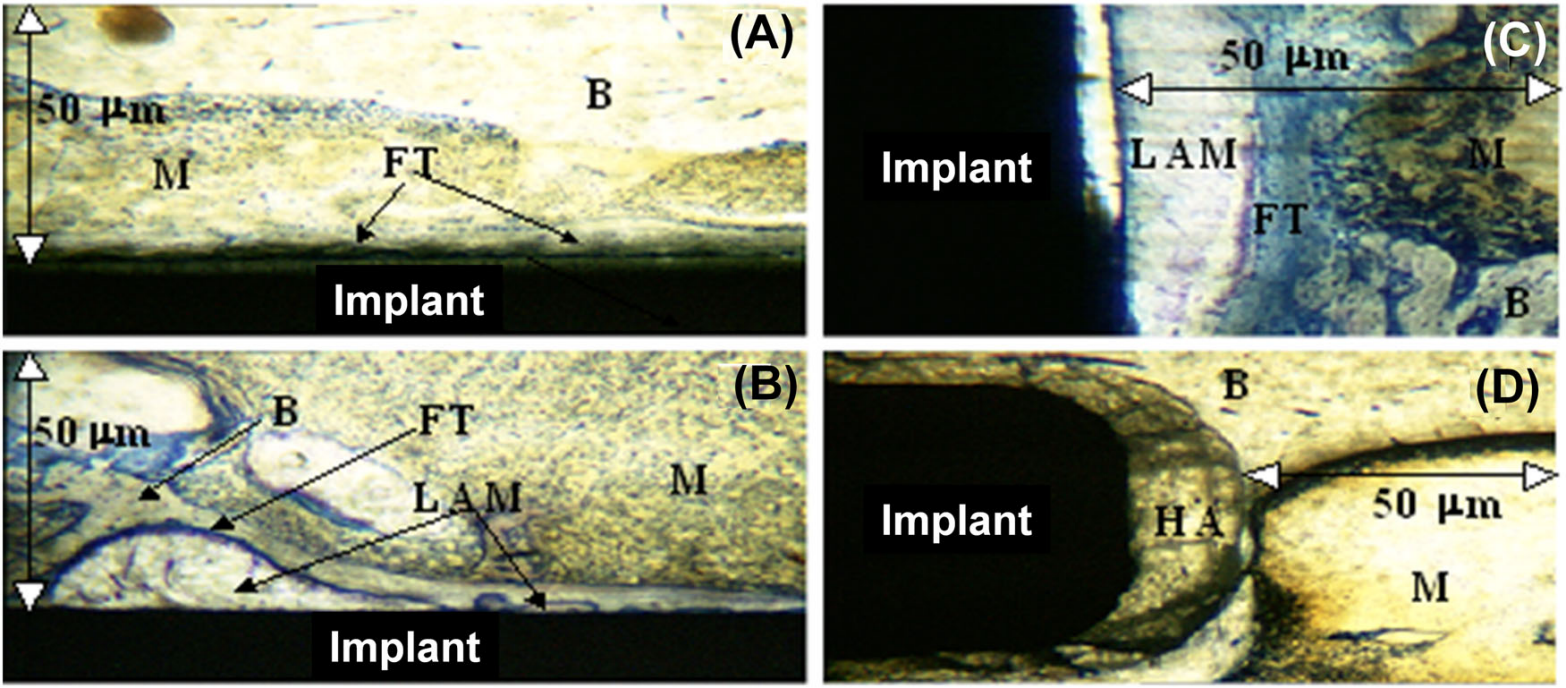
Typical experimental micrographs indicating different tissue types in the bone-implant interfacial area, where M is marrow, B is bone, FT is fibrous tissue and LAM is loosely associated matrix, for **a** stainless steel; **b**, **c** PC20; **d** hydroxyapatite (HA)

Figure 7b indicates the amount (percentage) of the different tissue types in the bone-implant interfacial area defined in Fig. 2c. The data complements that described in Fig. 7a, however, in this instance bone and marrow, significantly (*p* < 0.05) dominate over fibrous tissue and LAM formation for all surface coatings. For PC6, PC0 and SS there was significantly (*p* < 0.05) more fibrous tissue than LAM. In a similar manner to the implant interface, Fig. 7b indicates that HA had, significantly (*p* < 0.05), the most bone and marrow apposition and the least fibrous tissue formation in comparison to the other four surfaces. PC0 had significantly (*p* < 0.05) more fibrous tissue than PC6. Analysis of these data shows that there is a trend for a linear increase in LAM with increasing CMA content (R^2^ = 0.9) in the implant interfacial area; a weak linear dependency of LAM with CMA content was also found at the implant interface (R^2^ = 0.7). Typical histological micrographs of the bone-implant interface are shown in Fig. 8. These show a thin layer of fibrous tissue between the implant and bone (Fig. 8a–c) with the exception of HA where the bone is directly apposed to the ceramic (Fig. 8d). The LAM is primarily seen adjacent to the PC coated implants (Fig. 8b, c).

## Discussion

### In vitro response

It has been well documented that non-charge modified phosphorylcholine surfaces (PC0) significantly reduce cellular adhesion compared to tissue culture plastic controls [7, 9, 32]. Within 1 min of implantation most biomaterial surfaces are coated with a protein film [44], which in an in vivo environment can comprise over 200 different proteins [45]. Proteins adsorb onto surfaces to minimise surface free energy. This can occur through protein conformational change, partial dehydration of the protein and surface, and the redistribution of surface charges [46]. Subsequent cellular interactions are largely governed by the nature and conformation of these adsorbed proteins whereby the adsorbed proteins can act as ligands for cell-surface receptors [47]. PC0 surfaces interact with proteins without inducing conformational shape changes in their structure resulting in a decrease in protein adsorption. It is thought that their highly hydrophilic phosphorylcholine zwitterionic head group (MPC monomer, Fig. 1) results in the formation of a hydration layer that allows proteins to interact with the surface reversibly whereby the hydrated layer limits the dehydration step involved in adsorption [48]. This zwitterionic group may also decrease protein adsorption through charge neutrality. The subsequent decrease in protein adsorption significantly reduces cellular adhesion, including monocytes, macrophages, fibroblasts and human granulocytes [49]. Such a response was also observed in HOb cells (Fig. 3), whereby there are significantly (*p* < 0.001) less HOb cells on PC0 at 6, 24 and 48 h compared to PC5, PC20 and tissue culture plastic. The lack of cell adhesion to PC0 can also be seen visually in Fig. 5.

Figure 3 indicates that over early time periods (up to 48 h) increasing CMA content increases the number of adherent HOB cells. This is in agreement with the findings of Rose et al [32] who showed that increasing CMA content in PC polymers increased cell adherence. The increase in cell attachment is likely to be caused by either the non-specific electrostatic interaction of the predominantly negative cells and the positively charged surface or through an increase in the number of cellular adhesive proteins adsorbed onto the surface of the PC polymer caused by the increase in CMA content. It is possible that both these mechanisms are running concurrently. Rose et al [32] also showed that the inclusion of CMA content in PC polymers resulted in an increase in protein adsorption compared to their non-charged counterparts and that specific proteins have a minimal CMA or charge content requirement. Such dependencies may help explain the results observed in Fig. 4a that indicate the effect of PC cationic charge on HOb cell proliferation up to 28 days.

Figure 4a shows that HOb cells attached to PC5 after 24 h are in a non-proliferate state. This is in stark contrast to the number of cells proliferating on both tissue culture plastic and PC20 up to 28 days. Generally, initial cell adhesion occurs in the first 30–120 min of cell-surface contact followed by a stronger attachment involving the secretion and development of ECM, cellular migration and growth [50]. The larger number of proliferating osteoblasts, at 48 h, on PC20 and the control in comparison to PC5 can also be seen visually in Fig. 5. This suggests that there is only a weak interaction formed between the PC5 surface and the osteoblast cells, reducing stimulation of the cells to produce ECM. The lack of ECM production is likely to cause the subsequent loss of cells by apoptosis, which is seen at the later time points (Fig. 4a). The weaker interaction between PC5 and the osteoblast cells is likely to relate to the charge sensitive adsorption of proteins. For example, PC5 may not contain enough positive charge to adsorb vitronectin or fibronectin, adhesive glycoproteins that are known to play a key role in the anchorage of osteoblasts [50].

The results for ALP production by the cells shown in Fig. 4b have been adjusted for the change in cell number in the cultures shown in Fig. 4a. ALP is an early marker of osteoblast differentiation [51] and is only up-regulated by osteoblast cells after proliferation has finished and prior to mineralisation of the ECM. Once mineralisation starts, ALP production ceases [52]. On PC20 and control substrates the osteoblasts continue proliferating throughout the culture period, as seen in Fig. 4a, and do not reach confluence, which would enable the onset of differentiation throughout the culture and increase ALP production. ALP production per thousand cells decreases over the first 2 days in these cultures as the newly divided cells are not producing ALP. Thereafter it is likely that groups of cells are showing increased ALP but not the proliferating cells in the culture; thus, the ALP activity per thousand cells does not show a marked change. On PC5 the ALP production remains constant over the initial 2 days as cell number does not increase after which the very low cell number and ALP production prevent an accurate measure of ALP production per thousand cells.

Evidence of mineral formation on PC20 is supported by Fig. 5, which shows that on both PC20 and tissue plastic control surfaces HOb cells have developed fully spread morphologies that contain deposits of calcium phosphate (Fig. 6), which increased with time, up to 28 days. However, the EDX spectra (insets of Fig. 6a-d) showed that the calcium phosphate (Ca:P) ratio of both PC20 and control was approximately 0.6, considerably lower than hydroxyapatite (the mineral phase of bone) which has a Ca:P ratio of between 1.5 and 1.67 [53]. A lower Ca:P ratio is indicative of calcium deficient mineral. Such a calcium deficient HA maybe the due to the presence of the cationic surface charge of the PC that may be preventing Ca^2+^ deposition into the mineral.

### In vivo response

Figure 7a, b shows the amount (percentage) of different tissue types at the bone-implant interface and interfacial area respectively. The results indicate that the HA coating creates excellent bone apposition against the implant, showing significantly (*p* < 0.05) more bone and marrow implant apposition compared with the other four substrates. The degree of this apposition can be seen in Fig. 8d and is in agreement with the literature [54, 55]. Hydroxyapatite (Ca_5_(PO_4_)OH) based coatings create an interactive bond between the bone and implant that is characterised by the presence of a thin calcium phosphate (apatite) layer, which forms early in the implantation process. Kokubo et al. [56] argue that this intermediate apatite layer allows for the preferential proliferation and differentiation of osteoblasts on its surface, stimulating the formation of new bone.

In contrast, the phosphorylcholine and the stainless steel implants are apposed with fibrous tissue and LAM (Figs. 7 and 8a–c). Fibrous encapsulation of so-called “bio-tolerant” polymeric and metallic implants at the bone-implant interface has been well documented and is a consequence of the foreign-body reaction [57, 58]. After the immediate protein adsorption that follows implantation, local tissue damage causes the recruitment of white blood cells, including macrophages, which subsequently bind to the surface of the implant. However, these phagocytic cells cannot ingest the implant and fuse to form giant cells, recruiting fibroblasts, which subsequently secrete collagen matrix resulting in the fibrous encapsulation of the implant [45]. Both SS and PC0 show significantly (*p* < 0.05) more fibrous tissue than any other tissue type (Fig. 7). This is at odds with work by Goreish et al. [7] who showed that intramuscular PC0 implants in the rabbit were surrounded by 40% less inflammatory cells than polyethylene controls, concluding that this was due to PC0’s well hydrated surface and passive interaction with the surrounding tissue. Implant location (bone is rich in pre-matrix producing cells that can differentiate into fibroblasts), the nature of the controls (polyethylene is known to elicit a high fibrous response and stainless steel (SS) 316 is reasonably “bioinert” in an osseous environment), as well as the different animal studies used may explain the observed differences.

The reasons for the effect of PC CMA content on bone and marrow apposition is also debatable. Cationic charge decreases fibrous tissue formation with a consequential increase in bone and marrow apposition suggesting that increasing surface charge increases osseous-implant integration (Fig. 7a). However, this increase is not linear with increasing CMA content and both charged polymers (PC6 and PC20) show similar amounts of both bone and marrow and fibrous tissue (Fig. 7). Furthermore, no significant difference in bone and marrow and fibrous tissue exist between the negative control (SS) and the two charged PC coatings. One might expect that increasing surface charge would increase fibrous tissue formation, due to surface charge aggravates the foreign body response. However, Rose et al. [32] showed that although the presence of surface charge in PC increases the number of attached fibroblast cells, unlike other cells (eg monocytes and granulocytes), the number of fibroblasts on all charged materials PC materials with up to 30% CMA was maintained at a constant level.

An explanation for the decrease in both bone and marrow apposition and fibrous tissue formation with increasing PC CMA content is the increase in LAM with increasing surface charge (Fig. 7a). LAM is shown visually in Fig. 8c, d and can be characterised as a fluid-containing capsule comprised of a few cells and loosely aggregated matrix surrounded by a fibrous membrane. No reference in the literature exists as to the formation of such tissue at the implant-bone interface, though such tissue may be described as lesions; zones of tissue which have impaired function as a result of damage or disease [59]. The correlation (R^2^) of increasing LAM formation with CMA content increases from 0.7 to 0.9 when examining the interfacial area (Fig. 7b) of the implant as apposed to the interface (Fig. 7b) and is due to LAM extending from the surface of the implant and occupying space (Fig. 8b, c). LAM formation maybe caused by the preferential attachment of inflammatory cells such as monocytes that may become over stimulated in the production of tumour necrosis factor (TNF) -α, a chemotactic pro-inflammatory cytokine that can cause the formation of necrotic tissue [60]. In addition, the increase in charge may also be acting on cells in such a way that it results in local cell apoptosis.

Rose et al. [32] has shown that a variety of different cells and proteins are affected by the inclusion of positive surface charges into PC polymers. However, it is not clear how selective this effect is in a multi-cellular in vivo environment. For example, fibroblast cells may preferentially attach to the PC surface over osteoblast cells. Furthermore, both osteoblasts and fibroblasts share the same precursors in the bone marrow and these surfaces may be inducing fibroblast differentiation over osteoblasts, resulting in a fibro-encapsulated sheath. In addition, pro-inflammatory cells may preferentially bind to the charged PC surfaces and induce cell death and the production of LAM [27]. A further experiment, which examines the effects of PC and modified PC surfaces in a multicellular environment, would be an interesting way to assess which cells are preferentially attaching to the polymer surfaces. Alternatively, LAM maybe caused by continuous micromotion of the implant due to the failure of early bone growth at the bone-implant interface, however this seems unlikely as such a response might be expected equally with PC0, PC6 and SS implants; a trend that was not observed in the data. The in vivo results for hydroxy-apatite coated implants clearly accords with the clinical utility of this material; however, the relatively poor outcome for PC0, PC6 and PC20 indicate that these materials would not be suitable as coatings for bone bonding applications. Phosphorylcholine-based coatings may however show greater stability in vivo and could be useful as drug delivery vehicles in situations where bone bonding is not required or be used where inhibiting tissue bonding would be useful for example with fracture fixation plates. We do not know whether PC coating was lost from the surface of the pins due to mechanical damage during insertion. This could be investigated in cadaveric models by inserting coated pins into the tibia, breaking open the bone and determining the uniformity of coating on the retrieved pins.

The results presented in this paper describe the response of bone cells and bone to PC and modified PC surfaces and also demonstrate that in vitro experiments are not always accurate predictors of the more complex in vivo response. It was hypothesised that due to the increased proliferation and differentiation of HOB cells on PC20, and the evidence that these cells were undergoing mineralisation (Figs. 3–6), that PC20 coated implants would stimulate an increase in bone-bonding compared with the lower and non-charged PC materials, whereby osteoblasts are actively attracted to the surface of the polymer and mineralise to produce a tight implant-bone interface. However, in this limited scope in vivo experiment, it was shown that this was not the case. Ideally, a full time-course experiment would be needed to properly address the evolution of the biological response; here we were limited to one time point and chose 14 weeks post-implantation to allow for a period of fuller integration with the implant, as previous work had seen some differences at just 6 weeks post implant [34]. In fact, as bone ingrowth appears to occur at an earlier stage, our model may have suffered from the potential for newly-synthesised bone to undergo subsequent bone resorption due to mechanical stress or excessive inflammation.

## Conclusions

Modifying phosphorylcholine surfaces by the addition of cationic charge alters the response of cells to these surfaces and could allow the delivery of drugs, including antibiotics, from PC implant coatings. However, our studies show that whilst increasing positive surface charge allowed increased osteoblast adhesion to the PC surface and stimulated increased cell differentiation and the production of calcium phosphate deposits in vitro, when implanted in bone charge modified PC materials stimulated the production of fibrous tissue and areas of loosely associated matrix around the implant. Despite this, non-charged phosphorylcholine polymers were tolerated in the osseous environment and could be used to coat orthopaedic devices, where bone bonding is not required, for the delivery of antibiotics in order to reduce surgical site infection.
